# Rifampin resistance and its fitness cost in *Riemerella anatipestifer*

**DOI:** 10.1186/s12866-019-1478-7

**Published:** 2019-05-23

**Authors:** Jiakai Sun, Dekang Zhu, Jinge Xu, Renyong Jia, Shun Chen, Mafeng Liu, Xinxin Zhao, Qiao Yang, Ying Wu, Shaqiu Zhang, Yunya Liu, Ling Zhang, Yanling Yu, Yu You, Mingshu Wang, Anchun Cheng

**Affiliations:** 1 Research Center of Avian Diseases, College of Veterinary Medicine, Sichuan, Agricultural University, Chengdu, 611130 Sichuan China; 2 Institute of Preventive Veterinary Medicine, Sichuan Agricultural University, Chengdu, 611130 Sichuan China; 3 Key Laboratory of Animal Disease and Human Health of Sichuan Province, Chengdu, 611130 Sichuan China; 4 Guizhou Animal Husbandry and Veterinary Research Institute, Guiyang, 550005 Guizhou China

**Keywords:** *Riemerella anatipestifer*, Rifampin resistance, *rpoB* mutant, Spontaneous mutation, Fitness cost

## Abstract

**Background:**

*Riemerella anatipestifer* (*R. anatipestifer*) is one of the most important poultry pathogens worldwide, with associated infections causing significant economic losses. Rifampin Resistance is an important mechanism of drug resistance. However, there is no information about *rpoB* mutations conferring rifampin resistance and its fitness cost in *Riemerella anatipestifer*.

**Results:**

Comparative analysis of 18 *R.anatipestifer rpoB* sequences and the determination of rifampin minimum inhibitory concentrations showed that five point mutations, V382I, H491N, G502K, R494K and S539Y, were related to rifampin resistance. Five overexpression strains were constructed using site-directed mutagenesis to validate these sites. To investigate the origin and fitness costs of the *rpoB* mutations, 15 types of *rpoB* mutations were isolated from *R. anatipestifer* ATCC 11845 by using spontaneous mutation in which R494K was identical to the type of mutation detected in the isolates. The mutation frequency of the *rpoB* gene was calculated to be 10^− 8^. A total of 98.8% (247/250) of the obtained mutants were located in cluster I of the rifampin resistance-determining region of the *rpoB* gene. With the exception of D481Y, I537N and S539F, the rifampin minimum inhibitory concentrations of the remaining mutants were at least 64 μg/mL. The growth performance and competitive experiments of the mutant strains in vitro showed that H491D and 485::TAA exhibit growth delay and severely impaired fitness. Finally, the colonization abilities and sensitivities of the R494K and H491D mutants were investigated. The sensitivity of the two mutants to hydrogen peroxide (H_2_O_2)_ and sodium nitroprusside (SNP) increased compared to the parental strain. The number of live colonies colonized by the two mutants in the duckling brain and trachea were lower than that of the parental strain within 24 h.

**Conclusions:**

Mutations of *rpoB* gene in *R. anatipestifer* mediate rifampin resistance and result in fitness costs. And different single mutations confer different levels of fitness costs. Our study provides, to our knowledge, the first estimates of the fitness cost associated with the *R. anatipestifer* rifampin resistance in vitro and in vivo*.*

**Electronic supplementary material:**

The online version of this article (10.1186/s12866-019-1478-7) contains supplementary material, which is available to authorized users.

## Background

*Riemerella anatipestifer* is a bacterial pathogen that infects ducks, geese, turkeys and other poultry. This pathogen can cause diseases characterized by serositis and sepsis, also known as duck infectious serositis, new duck disease and duck septicemia. The prevalence of the disease can lead to high mortality rates and significant economic losses [1].

Rifampin is a semi-synthetic rifamycin that is one of the most effective and broad-spectrum antibiotics against bacterial pathogens. The active site of this drug is the β subunit of the RNA polymerase encoded by the *rpoB* gene. It has been reported that 95% of rifampin resistance is related to a missense mutation in the *rpoB* gene of *M. tuberculosis* [2]. The earliest studies in *E. coli* found that the *rpoB* mutations were mostly concentrated in three clusters: I, amino acids 507–533; II, amino acids 563–572; and III, amino acid 687. These regions are called "RIF regions" and are also known as rifampin resistance-determining regions (RRDRs) [3]. Afterwards, new sites outside the RRDRs continued to be reported [4, 5]. With the successful resolution of the high-resolution crystal structure of RNA polymerase and the development of molecular modeling techniques, analysis of the biosynthetic RNA polymerase structure proved that rifampin binds tightly to the DNA channel and blocks the normal transcription process. It can only block transcription initiation and did not inhibit the extension process [6]. The DNA channel was provided by a crab-like structure composed mainly of a β subunit and a β’ subunit [7]. When a specific mutation occurred in the *rpoB* gene, the rifampin molecule might be unable to block transcription due to its inability to form tight molecular forces (such as hydrogen bonds or van der Waals forces), and thus, it is unable to exert a drug effect [8].

RNA polymerase (RNAP) is highly conserved in all prokaryotes and is directly involved in the transcription of all genes in the genome. Since the β subunit is a critical subunit of the transcriptional machinery, RNAP, the *rpoB* mutation will change the structure of the β subunit and even RNAP, resulting in damage to global transcription. Therefore, it will affect the physiological characteristics of the organism and generate fitness costs. Mutation of these resistance genes located on chromosomes often carries a certain degree of fitness costs and affects the basic physiological activities of the strains [9].

In summary, so far there has been no information on the mechanism of rifampin resistance in *R. anatipestifer*. Based on previous references and knowledge of *R. anatipestifer* resistance, our study suggests that rifampin resistance of *R. anatipestifer* isolates may be due to the *rpoB* gene mutations. We attempted to restore the mutant type of isolates under laboratory conditions by using ATCC 11845 as the parental generation to elucidate the origin of *R. anatipestifer* rifampin resistance. In this study, the rpoB protein sequences of 18 *R. anatipestifer* strains were compared, and we speculated and validated the mutation sites that might be related to rifampin resistance. We then isolated rifampin-resistant mutants from ATCC 11845 and analyzed a number of phenotypic characteristics of these mutants, including growth, growth in competition, nitroprusside sensitivity and hydrogen peroxide sensitivity. Finally, the fitness of the *rpoB* mutants was evaluated in vivo.

## Result

### Rifampin minimum inhibitory concentration of *R. anatipestifer* and comparison of *rpoB* sequences

Since Clinical and Laboratory Standards Institute (CLSI) does not have a definitive rifampin resistance breakpoint for *R. anatipestifer*, we defined the minimum inhibitory concentration (MIC) < 0.5 μg/mL as rifampin-sensitive in this study. The DNAMAN software was used to compare the *rpoB* gene sequences of the 18 *R. anatipestifer* strains. The results are shown in Table 1. The *rpoB* amino acid sequences of the ATCC 11845, RCAD0122, RCAD0125, and RCAD0134 strains were identical, and their rifampin MICs were all less than 0.5 μg/mL; therefore, they were all categorized as rifampin-sensitive. The remaining 14 *R. anatipestifer* isolates all had *rpoB* mutations, including nine types of point mutations V382I, H491N, R494K, G502K, T528I, S539Y, A930T, T937A, and A993T. The MICs of these 14 *R. anatipestifer* strains on rifampin varied widely, ranging from less than 0.5 μg/mL to more than 256 μg/mL. There were four mutations, T528I, A930T, T937A and A993T in the RCAD0150 strain that were also sensitive to rifampin. Therefore, we considered that the *rpoB* mutation sites in RCAD0150 had little contribution to rifampin resistance, and these sites would be used as mutation sites that have no significant effect on rifampin resistance during the analysis. Therefore, it was concluded that five amino acid differences were related to rifampin resistance, V382I, H491N, G502K, R494K, and S539Y.

**Table 1:** Rifampin MICs and rpoB amino acid differences in 18 *R. anatipestifer* strains

NO.	Strain	RIF MIC(μg/mL)	Amino acid change
1	ATCC11845	< 0.5	–
2	RCAD0122	< 0.5	–
3	RCAD0125	< 0.5	–
4	RCAD0134	< 0.5	–
5	RCAD0150	< 0.5	T528I,A930T,T937A,A993T
6	RCAD0121	1	V382I,H491N, A930T,T937A,A993T
7	RCAD0183	1	H491N
8	RCAD0131	2	H491N, A930T,T937A,A993T
9	RCAD0124	4	H491N, A930T,T937A,A993T
10	RCAD0142	4	H491N, A930T,T937A,A993T
11	RCAD0111	8	H491N, A930T,T937A,A993T
12	RCAD0123	8	H491N, A930T,T937A,A993T
13	RCAD0133	8	H491N, A930T,T937A,A993T
14	RCAD0127	16	G502K,S539Y
15	RA-CH-1	32	R494K, A930T,T937A,A993T
16	RCAD0147	256	R494K, A930T,T937A,A993T
17	RA-CH-2	> 256	R494K
18	RCAD0181	> 256	R494K

Different *rpoB* mutations in the isolates had different levels of resistance to rifampin. Strains containing the V382I or H491N mutation showed resistance levels of 1 to 8 μg/mL. There were two point mutations, G502K and S539Y, in RCAD0127, and the rifampin MIC was 16 μg/mL. Strains containing the R494K mutation showed higher levels of resistance, at least 32 μg/mL. One set of data was noteworthy: two strains of *R. anatipestifer*, CH-1 and RCAD0147, carried identical *rpoB* mutations, but rifampin resistance actually showed a difference of at least 8-fold. Based on existing reports and knowledge of *R. anatipestifer* [8], it was speculated that, in addition to *rpoB* point mutations, high resistance to rifampin by RCAD0147 might be caused by differences in strain background or drug efflux pumps.

### Construction of overexpression strains to verify the relationship between *rpoB* mutation and rifampin resistance

The effect of *rpoB* mutations on the resistance to rifampin was tested by constructing multiple *rpoB* overexpression strains, of which ATCC 11845, ATCC 11845-pLMF03 and ATCC 11845-pLMF03::*rpoB* served as three control groups to exclude the influences of the plasmid itself and other sequences, except the point mutation in the *rpoB* gene, on rifampin resistance.

The rifampin MICs of the overexpression strains are shown in Table 2. The three control groups showed no difference, while the rifampin MICs of the overexpression strains all increased to at least 128 μg/mL. The results indicated that these five *rpoB* point mutations indeed mediated rifampin resistance. At the same time, the overexpression strains were also tested against ampicillin, cefuroxime, erythromycin, aztreonam, ciprofloxacin, kanamycin, chloramphenicol, clindamycin, sulfamethoxazole, and vancomycin. The MICs showed that the resistance of five antibiotics, ampicillin, cefuroxime, erythromycin, ciprofloxacin and chloramphenicol, increased by at least 4-fold. Among them, ampicillin and cephalosporin resistance originated from the resistance marker on the shuttle plasmid pLMF03.

**Table 2:** Determination of eleven antibiotics MICs of overexpression strains carrying different *rpoB* point mutations

Strain	Rifampin (μg/mL)	Ampicillin (μg/mL)	Cefuroxime (μg/mL)	Erythromycin (μg/mL)	Aztreonam (μg/mL)	Ciprofloxacin (μg/mL)	Kanamycin (μg/mL)	Chloramphenicol (μg/mL)	Clindamycin (μg/mL)	Sulfamethoxazole (μg/mL)	Vancomycin (μg/mL)
ATCC11845	< 0.25	< 0.25	0.5	< 0.25	4	< 0.25	128	4	< 0.25	256	32
ATCC11845-pLMF03	< 0.25	128	> 256	< 0.25	4	< 0.25	256	4	< 0.25	256	32
ATCC11845-pLMF03::*rpoB*	< 0.25	128	256	< 0.25	4	< 0.25	128	4	< 0.25	256	32
ATCC11845-pLMF03::*rpoB*(V382I)	256	32	128	8	4	4	256	16	0.5	256	32
ATCC11845-pLMF03::*rpoB*(H491N)	128	64	256	8	8	8	256	32	1	256	32
ATCC11845-pLMF03::*rpoB*(R494K)	128	128	> 256	8	8	8	256	32	1	256	32
ATCC11845-pLMF03::*rpoB*(G502K)	128	128	> 256	8	8	8	256	32	1	256	32
ATCC11845-pLMF03::*rpoB*(S539Y)	128	64	256	8	8	8	256	32	1	256	32

### Spontaneous mutation experiments

**Fig. 1:**
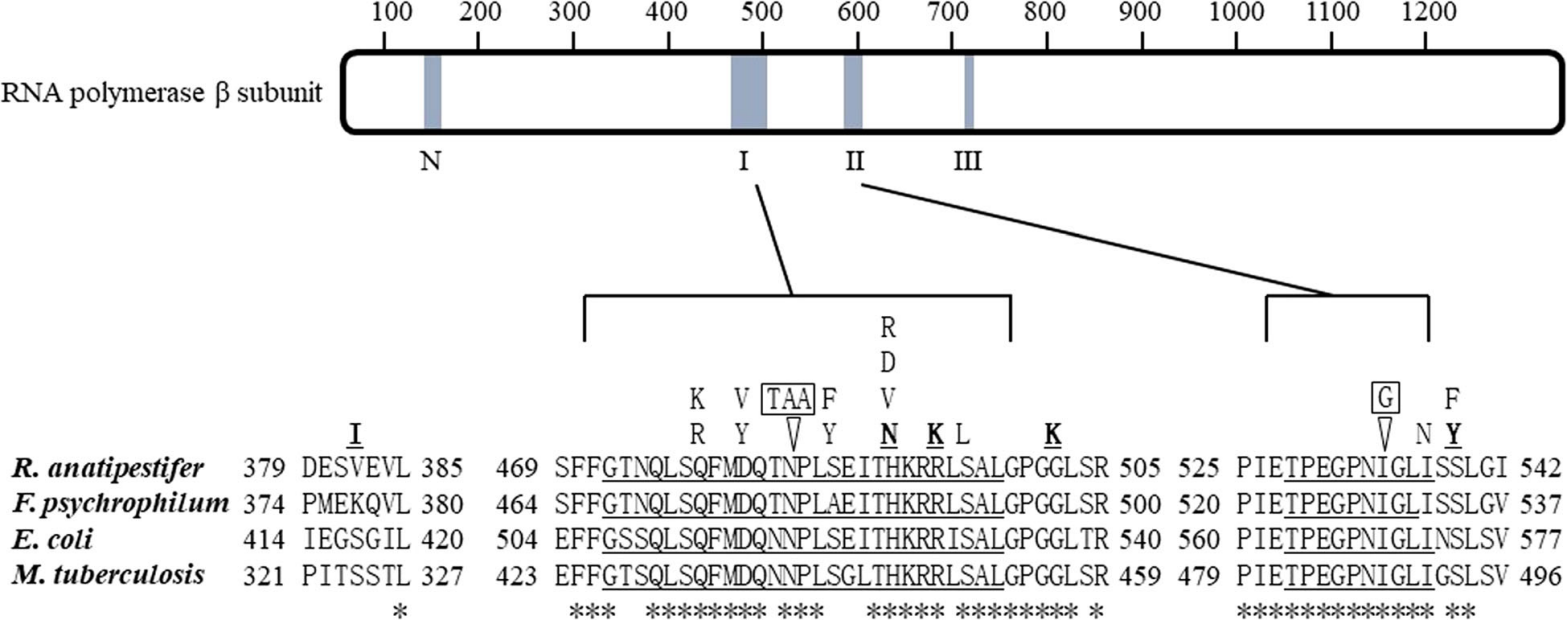
A schematic representation of the *rpoB* gene which encodes the β subunit of RNAP is shown (adapted from Jin DJ al. [3]). The shaded region on the β subunit is the RRDRs. The amplified portion is the region of the mutation involved in this study. The sequence consists of amino acid sequences of *R. anatipestifer*, *F. psychrophilum*, *E. coli* and *M. tuberculosis*, with the region RRDRs underlined. The asterisk at the bottom indicates that the corresponding amino acid at the top is conserved in the four strains. The mutation types associated with rifampin resistance in this study are listed above the sequence. The type identified in the isolates is underlined. The rest are spontaneous mutation types, and insert mutation types are framed. R494K was shared by both

The accurate rifampin MIC of ATCC 11845 was 0.004 μg/mL using the microdilution method. In addition, the number of viable cells per milliliter at an optical density 600 nm of 1.0 was approximately 2 × 10^9^ CFU. The spontaneous mutation strains from ATCC 11845 were screened at rifampin concentrations of 0.02 μg/mL, 0.04 μg/mL, 0.1 μg/mL, 0.2 μg/mL, 0.5 μg/mL, and 1 μg/mL. The results are shown in Table 3. The mutation frequency at 0.02 to 0.5 μg/mL rifampin was approximately 10^− 8^. When the concentration reached 1 μg/mL, the mutation frequency was reduced to 0. Next, we sequenced the RRDRs of the *rpoB* gene in 250 mutants isolated at a concentration of 0.2 μg/mL rifampin and found that 99.2% (248/250) of the mutants had only one point mutation. The statistics of these mutation types and mutation frequencies are shown in Table 4. There were 15 mutation types in these mutants, involving ten site changes, including 13 point mutations and 2 insertion mutations. Homology analysis found that up to 98.8% (247/250) were within *rpoB* cluster I. For the convenience of the analysis, we plotted all the *rpoB* gene mutation types in this study in Fig. 1, and also listed the rpoB protein sequences of *F. psychrophilum*, *E. coli* and *M. tuberculosis*. Compared with the mutation identified in the isolate, the mutation at position 494 was completely identical, and the frequency of this type was the highest in the spontaneous mutation experiment. In addition, mutations were also detected at positions 491 and 539, but they were not the same as those of isolates: at position 491, the codon CAT → AAT, and the changes in this position of the mutant involved three types, CAT→TAT or GAT or CGT; the 539 codon in the isolate was replaced by TCT → TAT, and the mutant was TCT → TTT. The 491 and 502 codon mutations were not found in the mutant strains.

**Table 3:** The mutation frequency of *rpoB* gene at different concentrations of rifampin

Strain	RIF concentration (μg/mL)	CFU/OD	Mutation frequency (×10^−8^)
ATCC 11845	0.02 (5 × MIC)	182.75	9.14
ATCC 11845	0.04 (10 × MIC)	173.25	8.66
ATCC 11845	0.1 (25 × MIC)	146.75	7.34
ATCC 11845	0.2 (50 × MIC)	138.50	6.93
ATCC 11845	0.5(125 × MIC)	20.50	1.03
ATCC 11845	1 (250 × MIC)	0	0

**Table 4:** Spontaneous mutation obtained from 0.2 μg/mL of rifampin in *rpoB* gene of 250 rifampin-resistant *R. anatipestifer*

Codon mutation	Nucleic acid change	No. of strains	Frenquency (%)	RRDRs	MIC (μg/mL)	MIC fold change
Q478R	CAG → CGG	24	9.6	I	128	32,000
Q478K	CAG → AAG	3	1.2	I	128	32,000
D481V	GAT→GTT	19	7.6	I	128	32,000
D481Y	GAT→TAT	6	2.4	I	8	2000
S487Y	TCT → TAT	2	0.8	I	128	32,000
S487F	TCA → TTA	14	5.6	I	128	32,000
H491Y	CAT→TAT	30	12	I	128	32,000
H491D	CAT→GAT	2	0.8	I	64	16,000
H491R	CAT→CGT	1	0.4	I	128	32,000
R494K	AGA → AAA	110	44	I	128	32,000
S496 L	TCA → TTA	36	14.4	I	128	32,000
I537N	ATT → AAT	1	0.4	II	0.5	125
S539F	TCT → TTT	2	0.8		0.5	125
485::TAA	::TAA	1	0.4		128	32,000
S496 L+ 535::G	::G	1	0.4		128	32,000
Total		252^a^	100.8^a^			

^a.^the reason why strain numbers are greater than the total number of mutants (250) and the frequencies are greater than 1 is that the presence of two double mutant strains

### Evaluation of rifampin-resistant spontaneous mutants in vitro

#### Growth curve

**Fig. 2:**
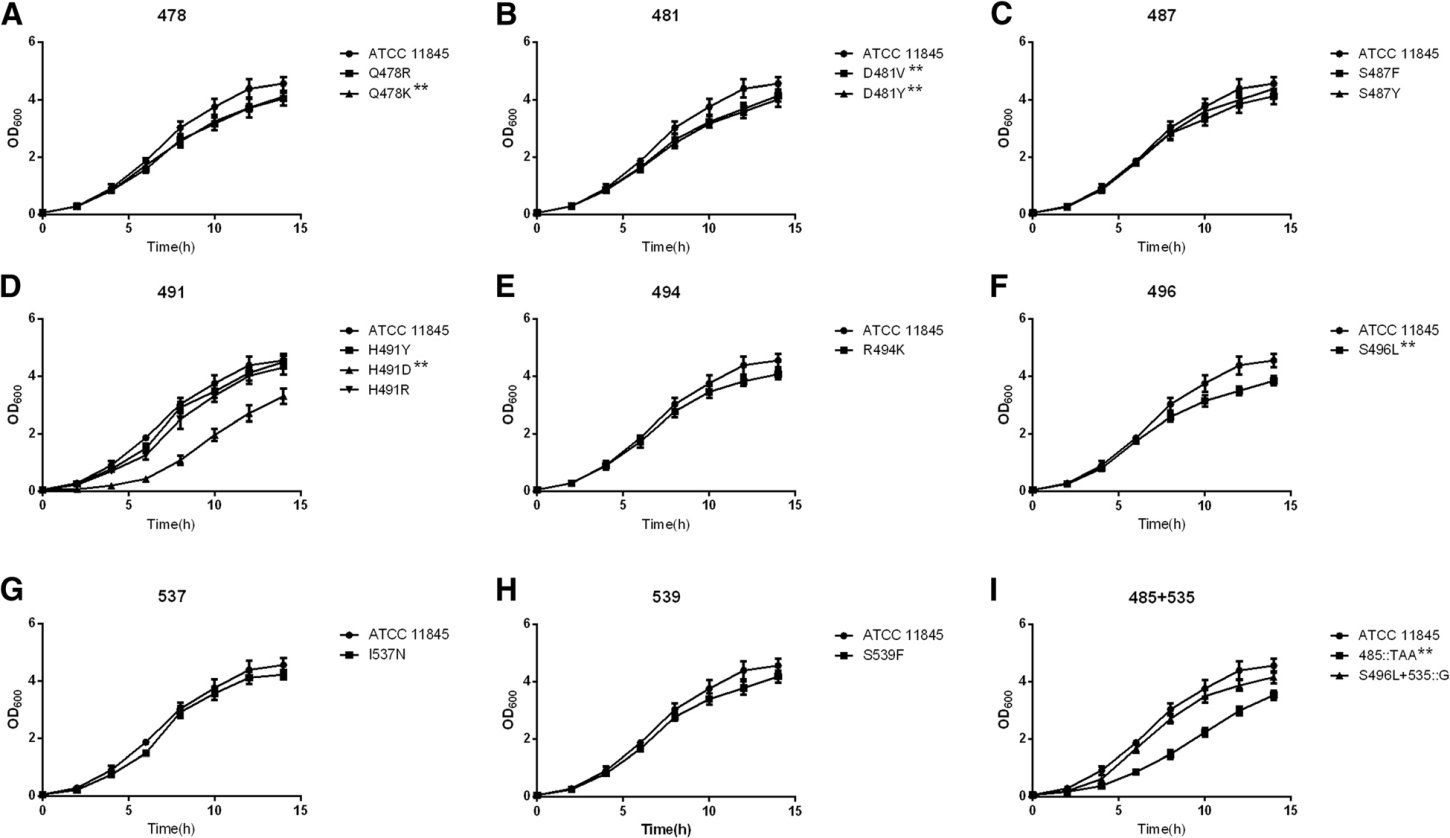
Growth curves of the spontaneous mutant strains and the parental strain. (A~H) Different mutation types at the same locus were plotted on the same graph. (I) Two insertion mutation types were plotted on the same graph. Error bars represent the standard deviation of three independent experiments

In order to evaluate the growth performance of spontaneous mutants in vitro, we plotted the growth curves when they were cultured alone. For the convenience of the analysis, the growth curves of different mutation types at the same site were plotted in the same figure, and the results are shown in Fig. 2. Compared with the parental strain, the growth rate of some mutant strains slowed down. Among them, the growth of H491D and 485::TAA were delayed most obviously.

#### Competitive experiments

In general, resistance mutations on chromosomes are accompanied by a certain degree of fitness costs. In this experiment, we investigated the differences in the competitiveness of the mutant and parental strains in vitro. The data were statistically analyzed according methods from the literature [10–12], and the results are shown in Table 5. This result was similar to that of the individual growth performance, and most mutant strains had similar growth abilities to their parental strains. The relative fitness of the 10 types of mutations was more than 0.9, the relative fitness of the 3 types of mutations was slightly reduced, the size ranged from 0.8 to 0.9, and two types of mutations (H491D and 535::G) were severely impaired in fitness and decreased to approximately 0.6. Combined with the growth characteristics of each strain, the mutants can be roughly divided into three categories: (i) the strains whose growth was similar to the parental strain and with low fitness cost, such as R494K and H491Y; (ii) the strains whose growth was similar to the parental strain and had a certain fitness cost, such as S496 L +  535::G; (iii) the strains whose growth was suppressed with higher fitness cost, such as H491D and 485::TAA. The results of this experiment also showed that different types of *rpoB* mutations conferred varying degrees of fitness costs to *R. anatipestifer*, and there was also a large difference in the fitness of mutation types produced by the same amino acid site.

**Table 5:** Fitness cost of investigated spontaneous mutation

Mutant	No.of generation		Cost per	D_0–1.0OD_	Relative fitness
Strain	g_S_	g_R_	generation		(g_R_/g_S_)
Q478R	8.76	7.88	0.08 ± 0.05	−0.08 ± 0.05	0.90 ± 0.06
Q478K	8.59	8.36	0.02 ± 0.03	−0.02 ± 0.04	0.97 ± 0.05
D481V	8.35	8.27	0.01 ± 0.05	−0.01 ± 0.05	1.00 ± 0.07
D481Y	10.51	9.50	0.07 ± 0.01	−0.07 ± 0.01	0.90 ± 0.02
S487Y	8.84	8.00	0.07 ± 0.05	−0.07 ± 0.05	0.91 ± 0.06
S487F	8.02	7.67	0.03 ± 0.02	−0.03 ± 0.02	0.96 ± 0.03
H491Y	8.93	8.51	0.03 ± 0.05	−0.04 ± 0.05	0.95 ± 0.07
H491D	9.08	5.47	0.35 ± 0.04	−0.44 ± 0.07	0.60 ± 0.02
H491R	8.35	7.32	0.09 ± 0.02	−0.10 ± 0.02	0.88 ± 0.03
R494K	10.00	9.13	0.07 ± 0.06	−0.07 ± 0.07	0.91 ± 0.07
S496 L	8.76	8.39	0.04 ± 0.04	−0.04 ± 0.04	0.95 ± 0.06
I537N	8.59	7.13	0.13 ± 0.04	−0.14 ± 0.04	0.83 ± 0.04
S539F	8.76	7.92	0.08 ± 0.01	−0.09 ± 0.14	0.91 ± 0.15
485::TAA	9.22	5.83	0.35 ± 0.05	−0.43 ± 0.08	0.63 ± 0.06
S496 L+ 535::G	9.71	8.01	0.14 ± 0.07	−0.15 ± 0.08	0.83 ± 0.08

#### Sodium nitroprusside and hydrogen peroxide sensitivity experiments

**Fig. 3:**
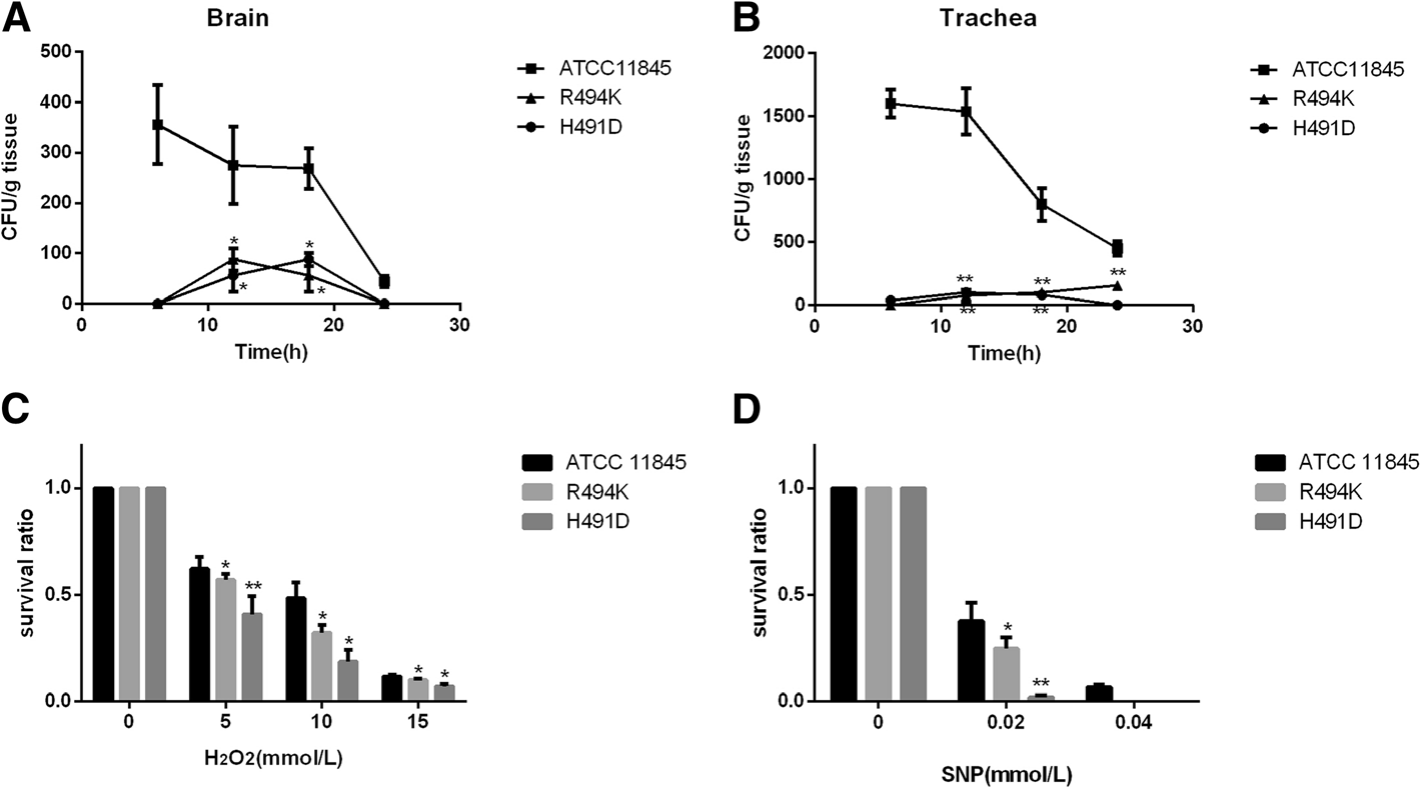
In vitro evaluation of the susceptibility of the wild-type ATCC 11845, mutants R494K and H491D to oxidative damage and nitrification stress and their ability to colonize ducklings. **a** and **b** Colonization capacity of wild-type ATCC 11845 and the mutants R494K and H491D in the duck brain and trachea. Values are the means of four independent experiments, and the results are shown as the relative number of CFU per gram of organs. **c** Effect of R494K and H491D substitutions on the sensitivity to oxidative damage. **d** Effect of R494K and H491D substitutions on the sensitivity to nitrificative stress. When the SNP concentration reached 0.06 mmol/L, none of the three strains could grow. Data are shown as the mean and SDs from four independent experiments, each with triplicate samples. The statistical significance of all the above tests was evaluated by Student’s *t*-test. The asterisk represents statistical significance (*, *P* < 0.05; **, *P* < 0.01)

When evaluating the sensitivity of *rpoB* mutants to active oxygen and reactive nitrogen, two characteristic mutants were selected in this study: R494K, which had the highest mutation frequency and whose in vitro competition is similar to parental strain, and H491D, with low mutation frequency and whose in vitro competition was significantly reduced. At the same time, the parental strain ATCC 11845 served as a control. To determine the ability to resist reactive oxygen species, the parental and mutant strains were exposed to different concentrations of hydrogen peroxide. As shown in Fig. 3, the viability of the mutants the ability to resist oxidative damage slightly decreased. As the concentration of hydrogen peroxide increased, the survival ratio of the parental strain and mutant strains gradually decreased. When exposed to 15 mmol/L hydrogen peroxide, the survival ratios of mutants R494K and H491D both reduced to below 10%. SNP was used as a NO generator to study the ability of mutants to resist reactive nitrogen. Exposed to 0.04 mmol/L SNP, the mutants R494K and H491D were completely killed, while a small amount of the parental strain survived. Compared with the parental strain, the statistical difference was significant (*P* < 0.05).

#### Colonization experiment in vivo

The environment in vivo is more complex than that in vitro. It is still unknown whether the *rpoB* gene mutation in *R. anatipestifer* affects the viability of strains in vivo. From the results shown in Fig. 3, it could be concluded that the colonization ability of both mutant strains in the brain and trachea was reduced compared to the parental strain. Compared with the parental strain, the statistical difference was significant (P < 0.05). The number of colonizing bacteria in the parental strain reached a peak at 12 h and gradually decreased. The presence of the parental strain and mutant strains were also detected in the brain and trachea, but the number of colonizing bacteria in the parental strain was still much higher than that of the mutant strain, though it gradually decreased over time. In addition, the number of bacteria colonized in the liver, lung, brain, blood, and spleen was also examined after 24 h of inoculation. Since the number of viable cells was too small and the difference was not significant, the results are not shown.

## Discussion

In recent years, the drug resistance of *R. anatipestifer* has become increasingly serious, and various mechanisms of drug resistance have gradually been revealed, such as those for resistance to chloramphenicol [13], lincomycin [14], aminoglycosides [15] and rifampin [16]. However, the resistance mechanism of rifampin has not been reported. Among other bacteria, rifampin resistance mechanisms have been reported, including mutations in the *rpoB* gene encoding the drug target enzyme [3, 17], glycosylation, modification of the drug structure by ADP ribosylation or phosphorylation modification [18–20], and the efflux effect of the drug efflux pump [8]. Since no plasmids, transposons, or inactivating enzymes related to rifampin resistance were found in the *R. anatipestifer* strains we identified, our study focused on the effect of the *rpoB* gene mutations on rifampin resistance.

In this study, we verified the link between the *rpoB* genotype change and rifampin sensitivity by site-directed mutagenesis and explored the origin of the *rpoB* mutation. This study identified five *rpoB* mutations related to rifampin resistance in *R. anatipestifer* isolates, including V382I, H491N, R494K, G502K, and S539Y. The amino acid sites 491 and 494 of the rpoB protein in *R. anatipestifer* corresponded to the 526 and 529 homologous positions of the rpoB protein in *E. coli*, respectively, which are located within the RRDRs cluster I. It has been reported that the replacement of amino acid 526 by another small molecule amino acid would confer RNAP resistance to rifamycin [21], which was consistent with our experimental results. Studies suggested that the rifampin resistance phenotype depended on the type of amino acid mutation [22]: the histidine side chain carried a positive charge, whereas the aspartic acid carboxylate group had a negative charge, and the aspartic acid molecule was smaller. At the 529 position, although arginine and lysine carried similar charges at the end of the side chain, the substitution of lysine destroyed the hydrogen bonds between arginine and Asp516, which affected the normal structure of RNAP [23]. The mutation S539Y outside the RRDRs was mentioned in rifampin-resistant *Salmonella* [24]. The changes in the hydrophilicity and molecular size of serine and tyrosine would lead to changes in the natural structure of RNAP, which might be a reason why rifampin cannot be combined with RNAP. The remaining mutation types V382I and G502K have not been reported. The differences in valine and isoleucine were mainly the size of the molecule, and the differences in glycine and lysine were more remarkable. The sizes, hydrophobicity and charge properties were different. Additionally, the effects of these two point mutations on the structure of RNAP need to be further explored. Meanwhile, the MICs of overexpression strains showed that the resistance of five antibiotics, ampicillin, cefuroxime, erythromycin, ciprofloxacin and chloramphenicol, increased by at least 4-fold. In fact, partial diploids of the double *rpoB* gene in the same cell have been reported in actinomycetes [25]. The presence of the wild-type and mutant *rpoB* genes caused the bacteria to be resistant to rifampin while the secondary metabolites pathways was altered. And the expression of *rpoB* allele was different at different growth stages. So far, there is no reasonable explanation for the rise of erythromycin, ciprofloxacin and chloramphenicol resistance.

We tried to screen out the same spontaneous *rpoB* mutation by rifampin pressure in the laboratory to prove our conjecture that the use of rifampin enriched the resistant strains. The results, however, are not satisfactory. The differences in mutation types between isolates and spontaneous mutant are worth considering. Almost 50% of the *rpoB* mutations were strain-dependent, and the interaction of different types of *rpoB* mutations with other genomes would affect transcriptional levels, resulting in varying levels of fitness costs, and thus affecting the viability of mutant strains [26]. The resistance levels of most of the isolates were relatively low, but the overexpression strain ATCC 11845-pLMF03::*rpoB*^+^ constructed according to the type of isolates all reached 128 μg/mL, indicating that the level of drug resistance conferred by the *rpoB* mutation might be affected by the strain background. Second, from the perspective of biological evolution, the replacement of the *rpoB* gene in a strain was not only a mutation of one amino acid site. The amino acid H531 of the *rpoB* gene in *Pseudomonas aeruginosa* could generate both H531R and H531Y mutations; continuous screening from two mutations was able to get the same site of the new mutation type H531C, while H531C weakened part of the H531R and H531Y fitness cost [27]. And that indicated that mutation from the wild type to H531C required a step-by-step process. In addition, as environmental conditions could affect protein stability and activity, *rpoB* mutations might affect the function of RNAP at different temperatures [27, 28]. Therefore, the selected *rpoB* mutations were specific under certain environmental pressures [29, 30], which also provided an explanation for the differences in detected *rpoB* mutations. Above all, the spontaneous mutation test in this study was carried out on solid medium without antibiotics at 37 °C lacking clinically specific environmental pressures, making all mutations possible to survive, which had little to do with their respective fitness. Studies in *F. psychrophilum* suggested that strains under the rifampin pressure were prone to enrich more single-nucleotide polymorphism, which would be associated with weakened virulence [31]. Therefore, the types of *rpoB* mutations obtained from spontaneous mutation tests were various, while the types identified in isolates were relatively concentrated.

Due to the frequency of spontaneous gene mutation sites (the sum of different mutation types in the same locus) and the distribution, the frequency of *rpoB* mutations in cluster I was as high as 98.8%. These amino acid sites were 494 (44%), 496 (14.4%), 491 (13.2%), 478 (10.8%), 481 (10%) and 487 (6.4%). These classical rifampin resistance mutation sites have been reported in *E. coli* [3], *Neisseria meningitidis* [12], *Salmonella* [24], *M. tuberculosis* [32] and *Pseudomonas aeruginosa* [33]. The frequency in cluster II was only 0.4%. The type of spontaneous mutation outside RRDRs was S539F, and no obvious phenotype was detected except for low levels of rifampin resistance. It was worth noting that two new insertion mutations had been detected. One was the insertion of a stop codon TAA at the amino acid 485, and the other was the insertion of a base G at the amino acid 535. Their biological characteristics were mainly manifested in the impairment of fitness.

The resistance caused by gene mutations on the chromosomes are often accompanied by a certain degree of fitness costs. In the absence of antibiotics, resistant strains were at a disadvantage to sensitive strains. From this study, there was no direct relationship between the magnitude of the fitness cost of the mutation and the level of rifampin resistance. Except for H491D and 485::TAA, most of the mutants had no obvious damage to growth performance in vitro. Studies in *Neisseria meningitidis* indicated that the growth of mutation S487F at 37 °C was significantly inhibited and had a high fitness cost [12], but the same mutation in *R. anatipestifer* was more normal. As with the study in *M. tuberculosis*, the fitness costs of different *rpoB* mutations were different, and the relative fitness of the H491D mutant was always the lowest of all mutation types at this site (equivalent to H526D in this study) [32]. However, colonization experiments in ducklings showed that whether R494K, whose in vitro growth and competitive ability were similar to the parental strain, or H491D, whose growth and competition abilities decreased, both of their colonization abilities were deduced in vivo. This suggests that all of the *rpoB* mutants in the ATCC 11845 background in the natural environment might be at a disadvantage. In fact, the colonization abilities of ATCC 11845 and isolates were confirmed, which again suggested that both the background of the strains and the *rpoB* mutation could have a significant impact on the resistance and pathogenicity. Among the spontaneous *rpoB* mutants from *Acinetobacter baumannii* ATCC 17978, the mutants carrying substitutions at amino acids 522 and 540 all showed phenotypes of impaired movement and reduced virulence [34]. The transcriptome information of the mutants showed that the expression levels of the four coding transporters and metabolic enzymes in the mutants were directly related to the above phenotypes. The virulence genes of the mutant strain in this study were worth exploring at the transcription level.

Although both the parental and mutant strains were cleared at a very low level, the decrease in the viable count of the parental strain within 24 h was gradual, and the number of colonies was gradually reduced from 10^3^ CFU to 10^1^ CFU. However, there were two mutant strains that were both at very low levels. In particular, its colonization could not complete within the trachea, suggesting that the mutant strain did not seem to be able to fight clearance from the immune system. Studies in *E. coli* Δ*lon* strains suggested that *rpoB* mutants could reduce the expression of capsular polysaccharide synthesis genes in this strain. Capsular polysaccharides could help the cells to fight immune mechanisms in vivo [35]. Considering the crucial role of RNA polymerase in transcription, studying transcriptome changes in mutants would provide useful information. At the same time, we found that the ability of mutants to resist hydrogen peroxide and NO was decreased in an in vitro sensitivity experiment. This result also supported the results of colonization experiments in vivo. Both active oxygen and reactive nitrogen in vivo were the environmental pressures that the bacteria would encounter. The defense against oxidative stress was crucial for the survival of bacteria in the body [36]. So far, the *rpoB* mutant has not been characterized in detail and may lack sufficient attention. The lacking dose of rifampin in use and the residual drug in the environment may provide the *rpoB* gene with the stress condition required for spontaneous mutation. The fitness cost of *rpoB* mutation may be due to changes in gene transcription level, and transcriptome analysis of mutant strains will be of great value. Therefore, further investigation and evaluation are necessary.

## Conclusions

In conclusion, the *R. anatipestifer* rifampin resistance is mainly derived from the *rpoB* gene mutation, whose types may be related to the strain background and environment stress. The spontaneous mutation of *rpoB* gene is concentrated in the mutation type with low fitness cost. And different *rpoB* mutations confer different fitness costs. Our study provides, to our knowledge, the first estimates of the fitness cost associated with the *R. anatipestifer* rifampin resistance in vitro and in vivo.

## Methods

### Strains, plasmids, primers and culture conditions

The seventeen *R. anatipestifer* isolates were from sick ducks in large-scale duck farms in Sichuan, Guangdong, and Henan Provinces, China, and they were isolated and identified by our laboratory. Their complete genome DNA sequences have been submitted to the GenBank database of the National Center for Biotechnology Information. *R. anatipestifer* ATCC 11845 and *E. coli* ATCC 25922 were obtained from the American Type Culture Collection (ATCC). The primers used in this study are listed in the (Additional file 1: Table S1). The *E. coli*-*R. anatipestifer* shuttle plasmid pLMF03 was constructed by our laboratory and stored in *E. coli* DH5α [37]. When necessary, the medium was supplemented with ampicillin (Amp, 100 μg/mL), cefoxitin (Fox, 1 μg/mL), kanamycin (Kan, 40 μg/mL), polymyxin B (PB, 40 μg/mL), and rifampin (RIF, 1 μg/mL).

### Nucleotide and protein sequence analysis

Nucleotide and protein sequence alignments were performed using the software DNAMAN 8.0 (Lynnon-Biosoft, Ontario, Canada). The differences in the nucleotide and amino acid sites of the 18 *R. anatipestifer rpoB* genes are shown in Table 1.

### Antimicrobial susceptibility testing

MICs of all the *R. anatipestifer* strains for rifampin and other antibiotic were determined according to the Antibiotic Drug Sensitivity Test Protocol of CLSI [38]. *E. coli* ATCC 25922 was used as a quality control strain for all tests. All measurements were repeated in triplicate.

### Construction of overexpression strains

For the site-directed mutagenesis of the *rpoB* gene, we first cloned the full-length *rpoB* sequence of the rifampin-sensitive ATCC 11845. After double-digestion, it was ligated to the shuttle plasmid pLMF03. Then, according to the requirement of QuikChange® Lightning Site-Directed Mutagenesis Kit (Agilent Technologies; catalog no. 210518), after the synthetic mutation chain and *Dpn* I digestion of the plasmid template, the product was transformed into *E. coli* DH5α competent cells and spread onto ampicillin-containing blood agar. After 24 h, the single colony would be cultured once again. The plasmids were extracted and examined by sequencing the full-length *rpoB* gene to confirm whether site-directed mutagenesis was successfully conducted. Five shuttle plasmids containing the expected *rpoB* site-directed mutagens were then introduced into *R. anatipestifer* ATCC 11845 using natural transformation [39]. PCR was used to identify the cefoxitin resistance gene *cfxA* to confirm the introduction of the pLMF03::*rpoB*^+^. To make it easier to understand, all of the constructed site-directed mutagenesis plasmids were collectively referred to as pLMF03::*rpoB*^+^, and the single mutations are represented by the “plasmid + mutation type” notation, such as pLMF03-*rpoB*(R494K); the spontaneous mutant that would be mentioned later was directly represented by the mutation type, such as R494K mutation.

### Spontaneous rifampin-resistant mutant generation experiment

The rifampin-sensitive ATCC 11845 strain was inoculated into Tryptone Soy Broth (TSB) medium and cultured at 37 °C until they reached the logarithmic growth phase. Then, 100 μL of the bacterial solution was added to 10 mL of fresh TSB medium to continue the culture and subculture three times. Then, 1 mL of the OD_600_ = 1 bacterial solution was taken and diluted properly and then spread on blood agar that contained 0 μg/mL, 0.02 μg/mL, 0.04 μg/mL, 0.1 μg/mL, 0.2 μg/mL, 0.5 μg/mL, or 1 μg/mL rifampin. After 24 h, colonies that grew on the plates were counted. The experiment was repeated three times. All mutant strains obtained from Tryptone Soy Agar (TSA) plates containing 0.2 μg/mL rifampin were re-plated, and their rifampin resistance-determining regions were sequenced.

### Growth curve

The single colonies of the *rpoB* mutants and the parental strain were cultured on TSA or TSB at 37 °C in a 5% CO_2_ atmosphere. The OD_600_ of 1 mL of broth was measured for 2 h, and the OD readings were recorded. The growth curve of the two strains was plotted on the abscissa with the sampling time and the OD value on the ordinate. The resulting data were finally plotted as a growth curve using the GraphPad Prism 7.0 software.

### Competition experiment in vitro

To evaluate the fitness cost of the mutants in vitro, the difference in competition ability between the mutant strains and the parental strain under the condition of no antibiotic pressure was determined in this experiment. Mutants and parental strains were mixed and co-cultured competitively in antibiotic-free TSB medium at 37 °C. The initial and final broth dilutions were counted by the flat colony counting method on TSA plates with or without rifampin (0.2 μg/mL). The paired strains were mixed and counted at a low concentration (approximately 10^6^ CFU) of 1:1 and took approximately 8 h to grow to mid-logarithmic phase. To reduce the differences in the growth status of each strain, equal amounts of bacteria were preincubated in TSB at 37 °C for 8 h to ensure that all the bacteria were in good condition. The number of mutant colonies obtained from the plates containing rifampin and the number of parental strains were equal to the number of colonies without rifampin minus the number of mutant colonies. The experiment was repeated three times. The number of generations of mutant and parental strains in the mixed broth was calculated as described by Billington et al. [10]: g = (logB/logA)/log2, where A represents the colony number per milliliter at time zero, and B represents the number of CFU per milliliter at an optical density 600 nm of 1.0. The fitness between the paired competing strains was calculated according to the formula described by Sander et al. [11]. The function D^0–1.0OD^ described by the reference to Colicchio et al. indicated the differences in fitness between the competing strains. This function could be interpreted as the natural logarithm of the quotient of the growth rate of the competing strains. If D^0–1.0OD^ = 0, there is no difference in fitness between the two strains. If D^0–1.0OD^ < 0, the fitness cost of the mutant strain increased. If D^0–1.0OD^ > 0, the fitness cost of the mutant strain decreased [12]. The cost per generation (cpg) is calculated as cpg = 1-e^D0–1.0OD^.

### Sodium nitroprusside sensitivity experiment

This experiment was performed to detect the sensitivity of the *rpoB* mutant to NO. Sodium nitroprusside was used as a NO generator. ATCC 11845 was grown in TSB, while the *rpoB* mutant was grown in TSB supplemented with 1 μg/mL rifampin. When the bacteria reached an OD_600_ of 1.0, 0.5 mL of bacterial broth was spotted on TSA plates containing different concentrations of SNP (0, 0.02, 0.04 and 0.06 mmol/L) and then was grown overnight at 37 °C. At the same time, each dilution was also spotted on TSA medium containing 1 μg/mL rifampin to determine the number of viable cells. The experiments were performed in triplicate. The results and graphics were performed using GraphPad Prism software 7.0, and the significance of the data was analyzed by Student’s *t*-test.

### Hydrogen peroxide sensitivity experiment

This experiment was to detect the sensitivity of the *rpoB* mutant to H_2_O_2_. ATCC 11845 was grown in TSB, while the *rpoB* mutant was grown in TSB supplemented with 1 μg/mL rifampin at 37 °C with shaking until the OD_600_ was 1.0. Each strain was centrifuged at 5000 r/min for 5 min to collect the bacterial cells, and 5 mL of 1 × PBS resuspension solution was added separately. After measuring the optical density at 600 nm, different concentrations of H_2_O_2_ (0, 5, 10 and 15 mmol/L) were added to each bacterial broth sample, and the mixtures were incubated at 37 °C for 30 min. After proper dilution of the bacterial liquid, 100 μL of the bacterial liquid was spotted on TSA plates and then was grown overnight at 37 °C. The number of viable cells on the plate after 24 h were counted. The experiments were performed in triplicate. The results and graphics were performed using GraphPad Prism software 7.0, and the significance of the data was analyzed by the Student’s *t*-test.

### Colonization experiment in vivo

All experiments with animals in this study were performed in strict accordance with the recommendations of the Guide for the Care and Use of Laboratory Animals of the Ministry of Science and Technology of China. All animal procedures were approved by the Animal Ethics Committee of Sichuan Agricultural University. Cherry Valley duckings were abstained from the Duck Farm of Chengdu Grimaud Breeding Co Ltd. (China) and raised for the colonization assay. The colonization experiments in vivo were conducted using 48 one-day-old ducklings that were divided into three groups of 16 ducks. Each duckling was intramuscularly inoculated with approximately 10^9^ CFU of mutant and parental strains at five days old. At 6 h, 12 h, 18 h and 24 h after inoculation. The duckings were anesthetized with an overdose intravenous injection of sodium pentobarbital (100 mg/kg body weight). The samples, including the brains and tracheas of four ducklings in each group, were collected. After being weighed, the samples were ground and mixed with 1 × PBS in a ratio of 1:4. The mixtures were diluted 5 times and 25 times, respectively, and spotted on blood plates with Kan and PB or RIF, Kan and PB as required. The colony morphology on the plate was observed and counted after 24 h.

### Statistical analysis

The data of growth curves were analyzed using two-way ANOVA. The error bars represent the standard deviations of three independent experiments. The data of sensitivity experiments in vitro and colony tests in vivo were plotted using the GraphPad Prism 7.0 software, and the significance of the data was analyzed by Student’s *t*-test. The asterisk represents statistical significance (*, *P* < 0.05; **, *P* < 0.01).

## Additional file

Additional file 1: **Table S1.** Primers and descriptions used in this study (XLSX 10 kb)

## Abbreviations

Amp: Ampicillin
ATCC: American Type Culture Collection
CLSI: Clinical and Laboratory Standards Institute
Fox: Cefoxitin
H_2_O_2_: hydrogen peroxide
Kan: kanamycin
MIC: Minimum inhibitory concentration
PB: Polymyxin B
PCR: Polymerase chain reaction
RIF: Rifampin
RNAP: RNA polymerase
RRDRs: Rifampin resistance-determining regions
SNP: Sodium nitroprusside
TSA: Tryptone Soy Agar
TSB: Tryptone Soy Broth
